# Pneumonitis associated with pembrolizumab plus chemotherapy for non-squamous non-small cell lung cancer

**DOI:** 10.1038/s41598-023-30676-y

**Published:** 2023-03-06

**Authors:** Daichi Fujimoto, Satoru Miura, Keisuke Tomii, Hiromitsu Sumikawa, Kenichi Yoshimura, Kazushige Wakuda, Yuko Oya, Toshihide Yokoyama, Takashi Kijima, Tetsuhiko Asao, Motohiro Tamiya, Atsushi Nakamura, Hiroshige Yoshioka, Takaaki Tokito, Shuji Murakami, Akihiro Tamiya, Hiroshi Yokouchi, Satoshi Watanabe, Ou Yamaguchi, Ryotaro Morinaga, Takayuki Jodai, Kentaro Ito, Yoshimasa Shiraishi, Yoshihito Kogure, Ryota Shibaki, Nobuyuki Yamamoto

**Affiliations:** 1 Internal Medicine III, Wakayama Medical University, Wakayama, Japan; 2 Department of Internal Medicine, Niigata Cancer Center Hospital, 2-15-3, Kawagishi-Cho, Chuo-Ku, Niigata, 951-8566 Japan; 3 Department of Respiratory Medicine, Kobe City Medical Center General Hospital, Kobe, Japan; 4 Department of Radiology, National Hospital Organization Kinki-Chuo Chest Medical Center, Sakai, Japan; 5 Medical Center for Translational and Clinical Research, Hiroshima University Hospital, Hiroshima University, Hiroshima, Japan; 6 Division of Thoracic Oncology, Shizuoka Cancer Center, Shizuoka, Japan; 7 Department of Thoracic Oncology, Aichi Cancer Center Hospital, Nagoya, Japan; 8 Department of Respiratory Medicine, Kurashiki Central Hospital, Kurashiki, Japan; 9 Department of Respiratory Medicine and Hematology, Hyogo Medical University, School of Medicine, Hyogo, Japan; 10 Department of Respiratory Medicine, Graduate School of Medicine, Juntendo University, Tokyo, Japan; 11 Department of Thoracic Oncology, Osaka International Cancer Institute, Osaka, Japan; 12 Department of Pulmonary Medicine, Sendai Kousei Hospital, Sendai, Japan; 13 Department of Thoracic Oncology, Kansai Medical University Hospital, Hirakata, Japan; 14 Division of Respirology, Neurology, and Rheumatology, Department of Internal Medicine, Kurume University School of Medicine, Kurume, Japan; 15 Department of Thoracic Oncology, Kanagawa Cancer Center, Yokohama, Japan; 16 Department of Internal Medicine, National Hospital Organization Kinki-Chuo Chest Medical Center, Sakai, Japan; 17 Department of Respiratory Medicine, National Hospital Organization, Hokkaido Cancer Center, Sapporo, Japan; 18 Department of Respiratory Medicine and Infectious Diseases, Niigata University Graduate School of Medical and Dental Sciences, Niigata, Japan; 19 Department of Respiratory Medicine, Comprehensive Cancer Center, Saitama Medical University International Medical Center, Saitama, Japan; 20 Department of Thoracic Medical Oncology, Oita Prefectural Hospital, Oita, Japan; 21 Department of Respiratory Medicine, Kumamoto University Hospital, Kumamoto, Japan; 22 Respiratory Center, Matsusaka Municipal Hospital, Matsusaka, Japan; 23 Department of Respiratory Medicine, Graduate School of Medical Sciences, Kyushu University, Fukuoka, Japan; 24 Department of Respiratory Medicine, National Hospital Organization Nagoya Medical Center, Aichi, Japan

**Keywords:** Non-small-cell lung cancer, Respiratory tract diseases, Risk factors

## Abstract

Studies elucidating detailed characteristics of pneumonitis in association with chemo-immunotherapy are limited. We aimed to investigate the characteristics of images, prognostic factors, and clinical course of combination therapy associated with pneumonitis. A multicenter, retrospective cohort study of patients with non-squamous non-small cell lung cancer who received a combination of platinum, pemetrexed, and pembrolizumab was conducted. Patients with confirmed pneumonitis established by an independent multidisciplinary team were enrolled. For 53 patients with pneumonitis, radiographic features at diagnosis predominantly comprised an organizing pneumonia pattern (62%, 33/53). Twelve (23%) patients experienced a worsening respiratory status during pneumonitis management, which was associated with a high mortality rate (58%, 7/12) during treatment. Severe grade at pneumonitis diagnosis (*p* < 0.001), diffuse alveolar damage (DAD) pattern (*p* = 0.002), and disease extent ≥ 25% in the lungs (*p* = 0.009) were significantly associated with worsening respiratory status. Furthermore, post-diagnosis survival was significantly worse in severe pneumonitis (*p* = 0.02) than in mild and in patients with the DAD pattern than in those without (*p* < 0.0001). We showed detailed clinical course of patients with pneumonitis and reported several important influencing factors. Given the small number of trials on pneumonitis, our findings provide valuable information to guide the development of appropriate management guidelines and improve pneumonitis treatment.

## Introduction

Lung cancer is the leading cause of cancer-related death worldwide^1^. Non-small cell lung cancer (NSCLC) accounts for approximately 80% of all lung cancers, and most NSCLC cases are unresectable and metastatic at initial diagnosis^2^. The development of immune checkpoint inhibitors (ICIs), such as programmed cell death protein 1 (PD-1)/programmed death ligand 1 (PD-L1) checkpoint inhibitors, has significantly changed the treatment strategy for NSCLC. The addition of the PD-1 inhibitor, pembrolizumab, to the combination of platinum and pemetrexed has recently become the standard first-line therapy for patients with untreated metastatic non-squamous NSCLC without a driver oncogene^3^.

ICIs can cause inflammatory side effects (immune-related adverse events [irAEs]) that differ from those related to other systemic therapies. Among severe irAEs, pneumonitis has been a frequent adverse event in prospective trials with ICIs, and it can be a potentially life-threatening irAE^4,5^. Real-world experiences suggest that pneumonitis has been the most common severe irAE in patients with lung cancer who receive these inhibitors^6,7^. Our previous report showed that the early incidence of pneumonitis associated with a combination therapy was much higher than that of pneumonitis associated with only cytotoxic chemotherapy^8,9^. Furthermore, pneumonitis can lead to worse survival outcomes in patients with advanced NSCLC who receive ICI monotherapy and combination therapy, as highlighted previously^9,10^.

In the clinical setting, we experienced heterogeneous clinical courses of pneumonitis, with some cases responding to treatment and improving, some not responding to treatment, and others improving but then re-worsening^9^. Regarding patient management, characteristics such as the extent of pneumonitis and radiological pattern have been considered in irAE management guidelines to facilitate the appropriate management of the disease^11,12^. However, few reports have described these characteristics, prognostic factors, and detailed clinical courses of pneumonitis associated with ICI treatment, including a combination therapy. This insufficient evidence impedes the improvement of care for patients who develop pneumonitis. Therefore, investigating detailed clinical courses and useful prognostic factors, including some of the characteristics outlined in the guidelines, is required to guide the future management of pneumonitis and improve patient outcomes.

This study aimed to investigate the characteristics of images, prognostic factors, and clinical course of pneumonitis associated with cytotoxic chemotherapy and pembrolizumab in patients with previously untreated non-squamous NSCLC in real-world settings.

## Results

### Patient characteristics

Among 299 patients who received the combination therapy, 53 with a confirmed diagnosis of pneumonitis were enrolled in this study. Patient characteristics are summarized in Table 1. The median age of the patients was 69.0 years, and there were 12 (23%) patients aged over 75 years. Most patients were men (79%), had a smoking history (86%), and had an Eastern Cooperative Oncology Group Performance Status score of 0 or 1 before pneumonitis (92%). Six (11%) and 35 patients (66%) had pre-existing interstitial lung disease and emphysema, respectively. Computed tomography (CT) revealed that all six patients with pre-existing interstitial lung disease had chronic fibrosing interstitial lung disease.

**Table 1:** Baseline patient characteristics at the diagnosis of pneumonitis.

	Overall (n = 53)
Age (years)	
Median (range)	69.0 (44–81)
Sex, n (%)	
Male	42 (79)
Smoking status, n (%)	
Current	23 (43)
Former	23 (43)
Never	7 (13)
ECOG PS score before pneumonitis, n (%)	
0	19 (36)
1	30 (57)
2	2 (4)
3	2 (4)
Stage, n (%)	
3	4 (8)
4	40 (75)
Recurrence after surgery	8 (15)
Recurrence after radiotherapy	1 (2)
PD-L1 TPS, n (%)	
≥ 50%	11 (21)
1–49%	18 (34)
< 1%	21 (40)
Not investigated	3 (6)
Pre-existing interstitial lung disease, n (%)	6 (11)
Emphysema, n (%)	35 (66)
Previous thoracic radiotherapy, n (%)	7 (13)

Abbreviations: *ECOG PS* Eastern Cooperative Oncology Group Performance Status, *PD-L1* programmed death ligand 1, *TPS* tumor proportion score.

### Pneumonitis features and clinical courses

The features and management of pneumonitis are summarized in Table 2. The median time to the onset of pneumonitis was 4.1 (95% confidence interval, 2.6–5.3) months. Radiographic features at the diagnosis of pneumonitis consisted predominantly of the organizing pneumonia (OP) pattern (62%), and half of the patients (51%) had an extent of pneumonitis of < 25%. At the diagnosis of pneumonitis, most patients (n = 43, 81%) were diagnosed with mild pneumonitis (Common Terminology Criteria for Adverse Events [CTCAE] grade ≤ 2) and the remaining patients (n = 10, 19%) were diagnosed with severe pneumonitis. Among the 27 (51%) patients who did not receive steroid therapy as the initial treatment, 13 received subsequent steroid treatment. In total, 39 patients were treated with steroid therapy, among whom 31 (79%) showed improvement. Among patients with mild (n = 43) and severe (n = 10) pneumonitis, 29 (67%) and 10 (100%) received steroid therapy, respectively. An improvement in pneumonitis was observed in 26 (90%) and 5 (50%) patients, respectively. One patient received the combination of high-dose steroid therapy and immunosuppressants but died due to infection without an improvement in respiratory status. The median time between the commencement of steroid use and the confirmation of improvement was 1.4 (95% confidence interval, 1.1–1.9) months.

**Table 2:** Clinical features and management of pneumonitis.

	Overall (n = 53)
CT pattern of pneumonitis	
Diffuse alveolar damage	4 (8)
Nonspecific interstitial pneumonia	5 (9)
Hypersensitivity pneumonitis	9 (17)
Organizing pneumonia	33 (62)
Simple pulmonary eosinophilia	2 (4)
Extent of pneumonitis	
< 25%	27 (51)
25–50%	17 (32)
> 50%	9 (17)
Initial CTCAE grade	
1	23 (43)
2	20 (38)
3	8 (15)
4	2 (4)
Worst CTCAE grade	
1	12 (23)
2	26 (49)
3	8 (15)
4	3 (6)
5	4 (8)
Worsening of CTCAE grade	17 (32)
Worsening of respiratory status due to pneumonitis	12 (23)
Initial treatment	
No steroid and immunosuppressive agents	27 (51)
High dose of steroid	16 (30)
Low dose of steroid	9 (17)
High dose of steroid + immunosuppressive agents	1 (2)
Clinical outcome	
Improved	45 (85)
Death due to infection during treatment	2 (4)
Death due to cancer during treatment	2 (4)
Death due to pneumonitis	4 (8)
Worsening of PS after the improvement*	8 (18*)

Abbreviations: *CT* computed tomography, *CTCAE* Common Terminology Criteria for Adverse Events.

*Among the 45 patients who improved.

We defined an increase in either oxygen supplementation or category of oxygen supplementation regardless of the presence or absence of treatment for pneumonitis as worsening of respiratory status. Twelve (23%) patients had worsening respiratory status due to pneumonitis during management. Most patients (85%, 45/53) achieved an improvement in pneumonitis; however, eight patients died (death due to infection (n = 2), cancer progression (n = 2), and pneumonitis (n = 4)) during the treatment. Among the patients who improved from pneumonitis (n = 45), eight (18%) had a worsening performance status. Regarding the category of oxygen supplementation for pneumonitis, seven patients required simple oxygen supplementation and two required a non-rebreather mask (NRB) at the diagnosis of pneumonitis. Six patients required simple oxygen supplementation, four required an NRB or high-flow nasal cannula (HFNC), and four died due to respiratory failure associated with pneumonitis at the worst status. Among the four patients who died due to respiratory failure associated with pneumonitis, all patients used an NRB or HFNC just before death.

### Patients with worsening respiratory status

A comparison of characteristics at the diagnosis of pneumonitis between patients with and without worsening of respiratory status is presented in Table 3. A severe CTCAE grade at the time of diagnosis of pneumonitis (*p* < 0.001), diffuse alveolar damage (DAD) pattern (*p* = 0.002), and the extent of pneumonitis > 25% in the lungs (*p* = 0.009) were significantly associated with a higher rate of worsening of respiratory status due to pneumonitis. Regarding the extent of pneumonitis, pneumonitis > 50% of the lungs was also associated with a significantly higher rate of worsening of respiratory status of patients due to pneumonitis (*p* = 0.02). More patients who developed worsening of respiratory status due to pneumonitis received steroids as the initial treatment than those without a worsening status (10/12 versus 16/41, *p* = 0.009). Based on these three factors, the clinical outcomes and courses of patients with pneumonitis are presented in Table 4 and Supplementary Fig. S1. The median time to onset of pneumonitis was significantly shorter in patients with worsening of respiratory status than in those without (2.0 months versus 4.8 months, *p* = 0.037). Among the patients who improved (n = 45), the rate of patients who had worsened performance status after the improvement of pneumonitis was significantly higher in patients who had worsened respiratory status than in those who did not (3/5 versus 5/40, *p* = 0.03).

**Table 3:** Comparison of characteristics at the diagnosis of pneumonitis in patients with and without worsening of respiratory status.

	Without worsening (n = 41)	With worsening (n = 12)	*p* value
Age (years)			0.76
Median (range)	69 (44–81)	67.5 (31–84)	
Sex, n (%)			0.051
Male	30 (73)	12 (100)	
Smoking status, n (%)			0.32*
Current	18 (44)	5 (42)	
Former	16 (39)	7 (58)	
Never	7 (17)	0 (0)	
ECOG PS just before pneumonitis, n (%)			0.07*
0	15 (37)	2 (17)	
1	24 (59)	7 (58)	
2	2 (5)	1 (8)	
3	0 (0)	2 (17)	
Initial CTCAE grade of pneumonitis			< 0.001*
1	22 (54)	1 (8)	
2	17 (41)	3 (25)	
3	2 (5)	6 (50)	
4	0 (0)	2 (17)	
CT pattern of pneumonitis			0.002*
DAD	0 (0)	4 (33)	
HP	6 (15)	3 (25)	
NSIP	5 (12)	0 (0)	
OP	28 (68)	5 (42)	
Simple PEo	2 (5)	0 (0)	
Extent of pneumonitis			0.009*
< 25%	25 (61)	2 (17)	
25–50%	12 (29)	5 (42)	
> 50%	4 (10)	5 (42)	
Pre-existing interstitial lung disease, n (%)	4 (10)	2 (17)	0.61
Emphysema, n (%)	25 (61)	10 (83)	0.18
Previous thoracic radiotherapy, n (%)	4 (10)	3 (25)	0.18

Abbreviations: *ECOG PS* Eastern Cooperative Oncology Group Performance Status, *CTCAE* Common Terminology Criteria for Adverse Events, *CT* computed tomography, *DAD* diffuse alveolar damage, *HP* hypersensitivity pneumonitis, *NSIP* nonspecific interstitial pneumonia, *OP* organizing pneumonia, *PEo* pulmonary eosinophilia.

*Comparison between smokers and never-smokers, performance status (PS) 0–1 and PS 2–3, grades 1–2 and grades 3–4, DAD and others, and ≥ 25% and < 25%.

**Table 4:** Clinical courses of patients who developed pneumonitis stratified by the extent of pneumonitis and the pattern of the image.

Pattern of the image	Extent of pneumonitis	N	Clinical course n (%)		
			Improvement (n = 45)	Worsening of respiratory status (n = 12)	Death during treatment (n = 8)
Mild pneumonitis at the diagnosis (n = 43)					
DAD (n = 1)	< 25%	0	–	–	–
	≥ 25%	1	0 (0%)	1 (100%)*	1 (100%)*
Non-DAD (n = 42)	< 25%	25	24 (96%)	1 (4%)*	1 (4%)*
	≥ 25%	17	16 (94%)	2 (12%)	1 (6%)
Severe pneumonitis at the diagnosis (n = 10)					
DAD (n = 3)	< 25%	0	–		
	≥ 25%	3	0 (0%)	3 (100%)**	3 (100%)**
Non-DAD (n = 7)	< 25%	2	1 (50%)	1 (50%)*	1 (50%)*
	≥ 25%	5	4 (80%)	4 (80%)*	1 (20%)*

Abbreviations: *DAD* diffuse alveolar damage.

*One patient develops worsening of respiratory status and dies during the treatment.

**Three patients develop worsening of respiratory status and die during the treatment.

### Survival and relapse

**Figure 1:**
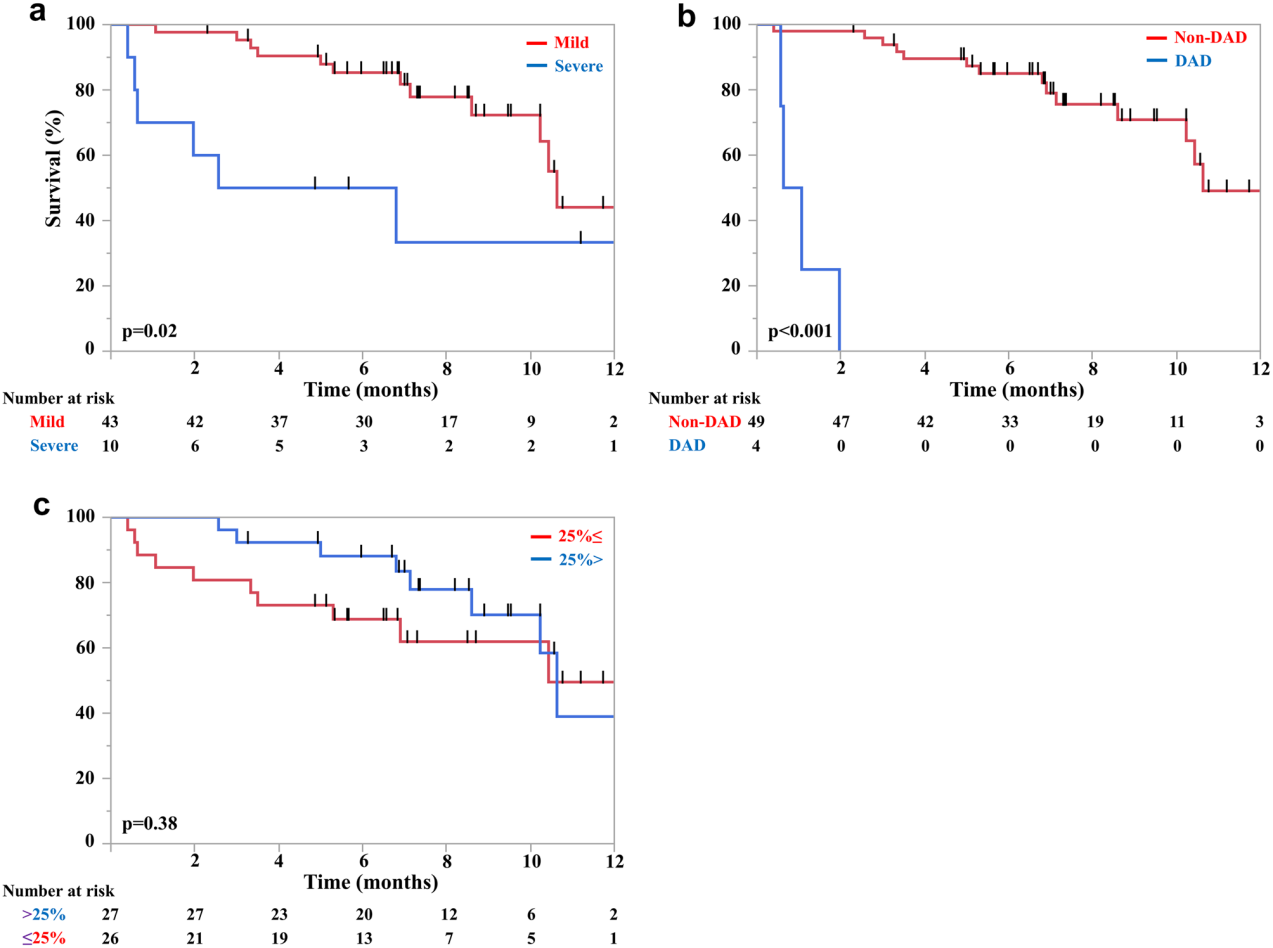
Kaplan–Meier survival curves of overall survival stratified by the severity of pneumonitis (mild or severe) (**a**) computed tomography image patterns (diffuse alveolar damage [DAD] or non-DAD) and (**b**) extent of pneumonitis at diagnosis (extent < 25% or ≥ 25%) (**c**).

During a median follow-up of 10.5 (range, 0.4–14.2) months after the diagnosis of pneumonitis, 18 overall survival events (34%) were observed. The survival curves after the diagnosis of pneumonitis are shown in Supplementary Fig. S2. The survival curves after the diagnosis of pneumonitis stratified by the severity of pneumonitis at the diagnosis, the extent of pneumonitis, and CT patterns of pneumonitis are shown in Fig. 1 and Supplementary Fig. S3. There were significant differences in the median survival after the diagnosis of pneumonitis between patients who developed mild and severe pneumonitis (10.5 versus 4.6 months, *p* = 0.02) and patients who developed non-DAD and DAD (10.5 versus 0.8 months, *p* < 0.0001). There was no significant difference in the median survival between patients who developed pneumonitis with extents of < 25% and ≥ 25% (10.5 versus 10.3 months, *p* = 0.38), and < 50% and ≥ 50% (10.5 months versus not reached, *p* = 0.38).

Two patients experienced relapsed pneumonitis after confirming the improvement in pneumonitis without rechallenging combination therapy. Both patients developed grade 2 (worst grade) pneumonitis at the first pneumonitis. Moreover, both patients with relapsed pneumonitis improved with steroid therapy. The periods between the start of steroids and the confirmation of improvement were 22 and 28 days, and the relapse occurred at 126 and 154 days, respectively, after the confirmation of improvement.

Eleven patients received rechallenge therapy, including: platinum agent, pemetrexed, and pembrolizumab (n = 2); platinum and pemetrexed (n = 1); pemetrexed (n = 3); pemetrexed and pembrolizumab (n = 4); and pembrolizumab (n = 1). All 11 patients recovered from mild first pneumonitis—grade 1 (n = 5) and grade 2 (n = 6). Of them, three patients experienced relapsed pneumonitis after the rechallenge. These three relapsed cases received pemetrexed and pembrolizumab (n = 2) and pemetrexed (n = 1). They all experienced mild relapsed pneumonitis; one patient developed grade 2 pneumonitis and two patients developed grade 1 pneumonitis.

## Discussion

In this study, we demonstrated the features and clinical courses of pneumonitis associated with cytotoxic chemotherapy and pembrolizumab using one of the largest cohorts of its type. Our study focused on the characteristics of pneumonitis described in the guidelines that have been underrepresented. We identified the heterogeneous clinical courses of pneumonitis as well as several features of pneumonitis that influence survival.

This study indicated the heterogeneous clinical courses of patients who developed pneumonitis. The CTCAE disease grade, image patterns, and extent of pneumonitis at onset were important factors that influenced the clinical course in our study. These factors were also associated with worsening respiratory status and worse prognosis after the diagnosis of pneumonitis. In fact, the American Society of Clinical Oncology irAE guideline recommends that the extent of pneumonitis be factored in^12^, and the European Society for Medical Oncology irAE guideline recommends that the pattern of pneumonitis be factored into the severity grade of the disease to manage patients treated with ICI therapy^11^. Further, we considered whether these factors were relevant to the success of the management plan. More patients with a < 25% disease extent tended to improve when therapy was withheld (9/15, 60%) than those with a disease extent of ≥ 25% (4/9, 44%). Withholding therapy is the treatment method suggested for patients with grade 1 pneumonitis in the European Society for Medical Oncology guidelines, regardless of the extent of pneumonitis. However, as suggested in the American Society of Clinical Oncology guidelines, we discovered that the extent of pneumonitis might be a useful marker for the treatment plan. As for the image patterns, all four patients with the DAD pattern died during treatment for pneumonitis regardless the CTCAE grade and extent of pneumonitis. Among patients with severe pneumonitis in our study, all three patients with the DAD pattern died during treatment. In contrast, only two of the seven patients with the non-DAD pattern died during treatment. Further, only one patient with CTCAE grade 1 died of pneumonitis, and the pattern of images had changed from the OP to the DAD pattern during follow-up. These results suggest that image patterns may also be important for the management plan, as in the European Society for Medical Oncology guidelines (despite not being included in the American Society of Clinical Oncology guidelines). No study has demonstrated the importance of these factors for managing pneumonitis associated with ICI therapy, even though the guidelines suggest that they should be considered—in addition to the CTCAE grade—when determining disease severity. Considering the respective guidelines and our results, a classification of disease severity that considers all these factors may be necessary for more appropriate treatment.

We also identified the devastating impact of worsening of respiratory status on survival due to pneumonitis. Seven out of 12 patients who developed a worsening of respiratory status died during the treatment for pneumonitis; conversely, only one died during the treatment for pneumonitis among the 41 patients who did not have a worsening of respiratory status. Additionally, the rate of patients who had worsening of performance status after the improvement of pneumonitis was significantly higher in patients who had worsening of respiratory status than in those who did not. Furthermore, we showed that the time to onset of pneumonitis was significantly shorter in patients with worsened respiratory status than in those who did not. Based on these results, patients with worsening of respiratory status may need to be considered for a more intense immunosuppressive approach, and more attention should be paid to early-onset pneumonitis.

Notably, we demonstrated the data of patients who experienced relapsed pneumonitis. Two patients experienced relapsed pneumonitis after confirming the improvement of pneumonitis without rechallenge combination therapy. Additionally, approximately 30% of the patients had relapsed pneumonitis after the combination therapy rechallenge. In our study, as stated in the irAE guidelines, all patients had mild cases of pneumonitis and were rechallenged after improvement^12^. Although the number of relapsed and rechallenged patients in our study was limited, these relapsed events may occur at a certain frequency. A large-scale study of relapsed cases and rechallenged cases is awaited.

The presence of a central review of the clinical and radiological description of patients who developed pneumonitis is considered a strength of this study. The CT images at baseline and the diagnosis of pneumonitis were evaluated by an independent multidisciplinary team without knowledge of the patient’s outcome. Subsequently, the diagnosis of pneumonitis was confirmed based on the clinical data and CT images. The most difficult patients to determine were those with a simple pulmonary eosinophilia (PEo) pattern (n = 2), which was difficult to differentiate from bacterial pneumonia that improved even without antimicrobial therapy. These patients in our study were considered to have drug-induced pneumonitis based on the clinical course after discussion. With the above high-quality judgment, all-grade pneumonitis occurred in 17.7% of our patients. These rates are significantly higher than those in the KEYNOTE-189 trial, where the frequency rate of all-grade pneumonitis was 4.4%^3^. However, previous studies have shown that the incidence rate of ICI-associated pneumonitis is significantly higher in real-world settings than in clinical trials, and Japanese people are susceptible to pneumonitis^7,13^. These might be the reasons for the discrepancy in the incidence rate between previous clinical trials and our study.

This study has several limitations**.** First, it was a multicenter retrospective study with not large sample size. Although this study was conducted based on the central determination of pneumonitis, not all patients underwent the same tests and uniform diagnostic criteria for pneumonitis were not established and applied. Only prospective studies on drug-induced pneumonitis conducted under a uniform definition of the diagnosis could solve this issue. Second, this study included small subsets of patients, such as relapsed cases, all patients had non-squamous NSCLC, and almost all patients were men and belonged to a single ethnicity (Japanese).

In conclusion, this study demonstrated that the CTCAE grade of pneumonitis, the pattern of the images, and the extent of pneumonitis were important factors that influenced the clinical course. In addition, in this study, we showed that the worsening of respiratory status during the treatment was associated with a poor prognosis. Further, some patients experienced relapsed pneumonitis during the hold of the therapy or after the rechallenge. Given the small number of trials for pneumonitis, the results of this study will serve as a basis for conducting future studies to investigate treatment strategies for these patients and consider more appropriate management guidelines.

## Methods

### Study design and patients

This was a multicenter, retrospective, hospital-based cohort study of consecutive patients with chemotherapy-naive advanced non-squamous NSCLC who received pembrolizumab, a platinum agent, and pemetrexed at one of 36 hospitals in Japan between December 2018 and June 2019. Clinical data of each patient were extracted from medical charts and entered into a database.

This study cohort was created to conduct two primary evaluations. The first primary analysis aimed to investigate the early incidence of pneumonitis and its association with survival, as reported previously^9^. The present report is a second prespecified primary analysis to determine characteristics, prognostic factors, and clinical course of pneumonitis in patients who developed pneumonitis associated with combination therapy with updated data.

Patients aged older than 20 years were enrolled if they had pathologically confirmed metastatic non-squamous NSCLC without sensitizing epidermal growth factor receptor or anaplastic lymphoma kinase mutations and had received a combination of platinum, pemetrexed, and pembrolizumab (combination therapy) as the first-line treatment.

Smoking status was categorized as never (never smoked), current (smoked within 1 year of diagnosis), and former (other smoking status). PD-L1 expression was assessed using the PD-L1 IHC 22C3 pharmDx assay (Agilent Technologies, Santa Clara, CA, USA) and was categorized based on the tumor proportion score.

This study was approved by the Ethical Review Board or Institutional Review Board of each participating institute including Wakayama medical university Ethics Committee. This study was performed in accordance with the principles of the Declaration of Helsinki. We attained adequate consent for using electronic patient records through an opt-out strategy owing to the study’s retrospective nature. All images were obtained with informed consent (or formal waiver of consent) with approval by the Ethics Committee of our hospital. This study followed the Strengthening the Reporting of Observational Studies in Epidemiology (STROBE) reporting guideline for cohort studies.

### Pneumonitis

The diagnosis of pneumonitis was established and confirmed by a treating medical oncologist and an independent multidisciplinary team comprising two pulmonologists and a radiologist. The diagnosis was based mainly on clinical data and CT images before the combination therapy and during pneumonitis. Patients with a significantly high probability of an alternative diagnosis based on independent judgment, such as cancer progression, congestive heart failure, radiation pneumonitis, and lung infection, were excluded as per these data.

The grade of pneumonitis was determined by the treating pulmonologist or oncologist and two independent pulmonologists according to the CTCAE version 5.0. Mild and severe pneumonitis were defined as grade ≤ 2 and grade ≥ 3, respectively.

Initial treatment for pneumonitis was defined as steroid and immunosuppressive agents within 1 week after the development of pneumonitis. Steroid doses were expressed as prednisone equivalents. Additionally, we defined a high steroid dose as a prednisolone equivalent dose of ≥ 1.0 mg/kg^12^.

Worsening of respiratory status due to pneumonitis was defined as an increase in either oxygen supplementation or category of oxygen supplementation for pneumonitis regardless of the presence or absence of treatment for pneumonitis; additionally, this status was confirmed. The categories of oxygen supplementation were as follows: (1) no oxygen supplementation; (2) oxygen supplementation but not requiring HFNC or NRB (simple oxygen supplementation); (3) HFNC or NRB; (4) non-invasive positive-pressure ventilation; (5) intubation with mechanical ventilation; or (6) death due to respiratory failure associated with pneumonitis^14,15^.

Improvement of pneumonitis was defined as improvement in oxygenation, respiratory symptoms, and lung field shadowing in patients who received steroids at a prednisolone equivalent dose of ≤ 10 mg^11^.

Relapsed pneumonitis was defined as pneumonitis after the confirmation of improvement and before the start of new anticancer therapy other than pembrolizumab, a platinum agent, and pemetrexed. Relapsed pneumonitis cases were divided into pneumonitis before and after the rechallenge of combination therapy.

### Radiology

**Figure 2:**
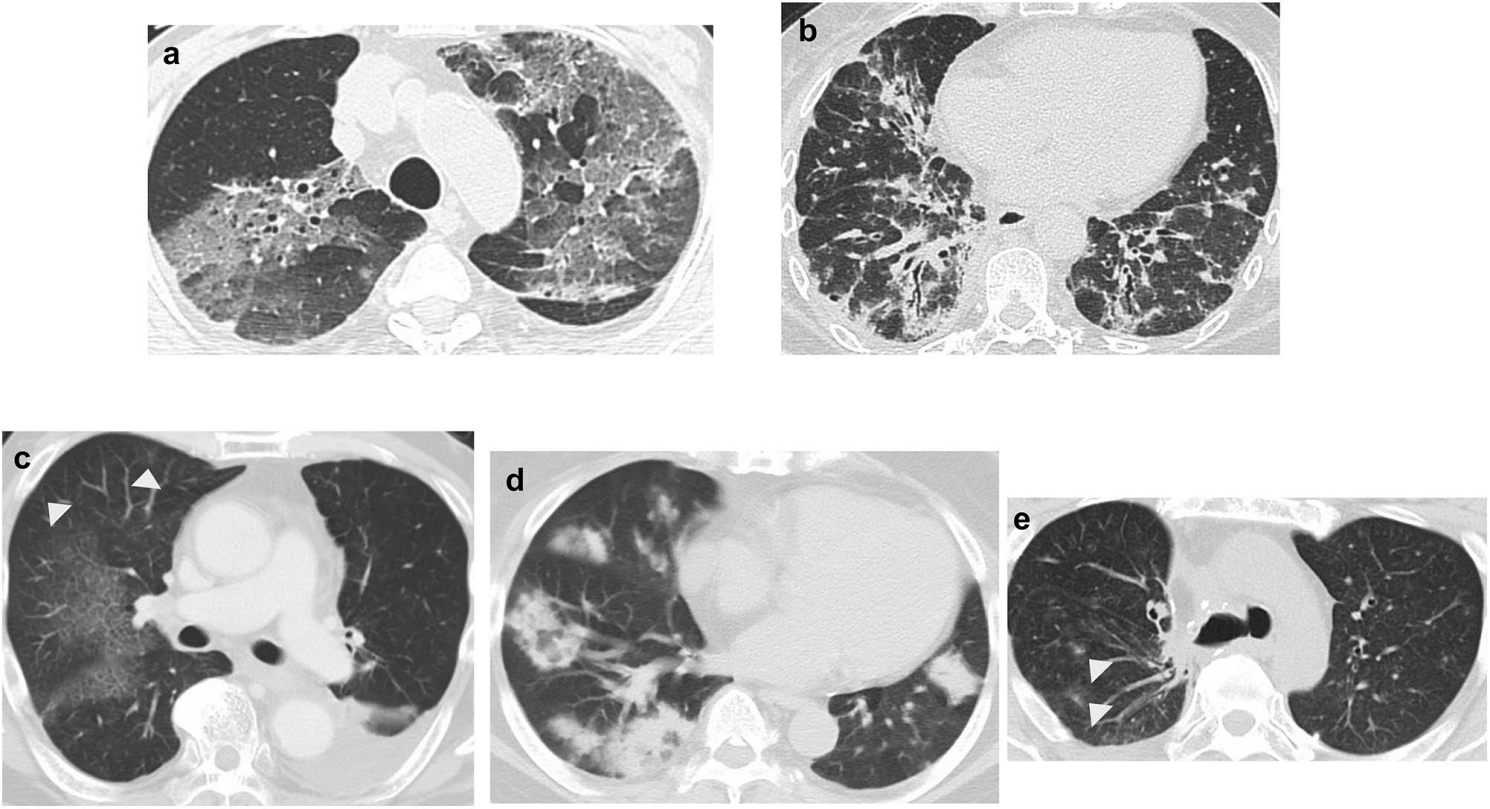
Drug-related pneumonitis showing new diffuse ground-glass opacities, consolidation, and traction bronchiectasis, indicative of a diffuse alveolar damage pattern (**a**). Drug-related pneumonitis showing new ground-glass opacities, irregular reticular opacities, and irregular reticular opacities with predominant lower lung involvement, indicative of the nonspecific interstitial pneumonia pattern (**b**). Drug-related pneumonitis showing new wide areas of faint ground-glass opacities with some patchy nodular lesions (arrowheads), indicative of the hypersensitivity pneumonitis pattern (**c**). Drug-related pneumonitis showing new ground-glass opacities and consolidations with multifocal distribution, indicative of the organizing pneumonia pattern (**d**). Drug-related pneumonitis showing new focal opacity areas (arrowheads). Lesions disappear only with withdrawal of drug therapy (not shown here), with features compatible with the simple pulmonary eosinophilia pattern (**e**).

Pre-existing interstitial lung disease, emphysema, pattern of pneumonitis, and extent of pneumonitis were determined by two experienced pulmonologists and an experienced radiologist based on CT findings. Pre-existing interstitial lung disease did not include radiation pneumonitis. In each case, CT patterns of pneumonitis were classified according to a previous report^15^ as (1) DAD pattern; (2) nonspecific interstitial pneumonia pattern; (3) hypersensitivity pneumonitis pattern; (4) OP pattern; and (5) simple PEo pattern^16^. Furthermore, we divided CT patterns of pneumonitis into DAD and non-DAD according to the irAE guideline^11^. The extent of pneumonitis was divided into < 25%, 25–50%, and > 50% of the lung parenchyma^12^, and the typical images of patients in our cohort are shown in Fig. 2.

### Statistical analyses

Age was compared using the Wilcoxon rank sum test. Dichotomous variables were analyzed using the chi-squared or Fisher’s exact test, as appropriate. The Kaplan–Meier method was used to estimate survival outcomes, and groups were compared using the log-rank test. A two-sided *p* value < 0.05 was considered statistically significant.

## Supplementary Information

Supplementary Figures.

## Data Availability

Research data will be shared upon reasonable request to the corresponding author.

## Abbreviations

DAD: Diffuse alveolar damage
NSCLC: Non-small cell lung cancer
ICI: Immune checkpoint inhibitor
PD-1: Programmed cell death protein 1
PD-L1: Programmed death ligand 1
irAE: Immune-related adverse event
CT: Computed tomography
OP: Organizing pneumonia
NRB: Non-rebreather mask
HFNC: High-flow nasal cannula
CTCAE: Common terminology criteria for adverse events
PEo: Pulmonary eosinophilia
