# Supplementary material for: Pneumonitis associated with pembrolizumab plus chemotherapy for non-squamous non-small cell lung cancer
Source: Sci Rep. 2023 Mar 6;13:3698. doi: 10.1038/s41598-023-30676-y (PMC9988982; doi:10.1038/s41598-023-30676-y)
Supplement: Supplementary file 1 — Supplementary Figures. [file 41598_2023_30676_MOESM1_ESM.pdf]

Supplementary Figure S1. Clinical courses of patients who developed grades 1 (a), 2 (b), 3 (c), and 4 (d) pneumonitis at the diagnosis of pneumonitis. These are stratified by the extent of pneumonitis at diagnosis (extent <25% or ≥25%) and the computed tomography image pattern (diffuse alveolar damage [DAD] or non-DAD).

Supplementary Figure S1a.

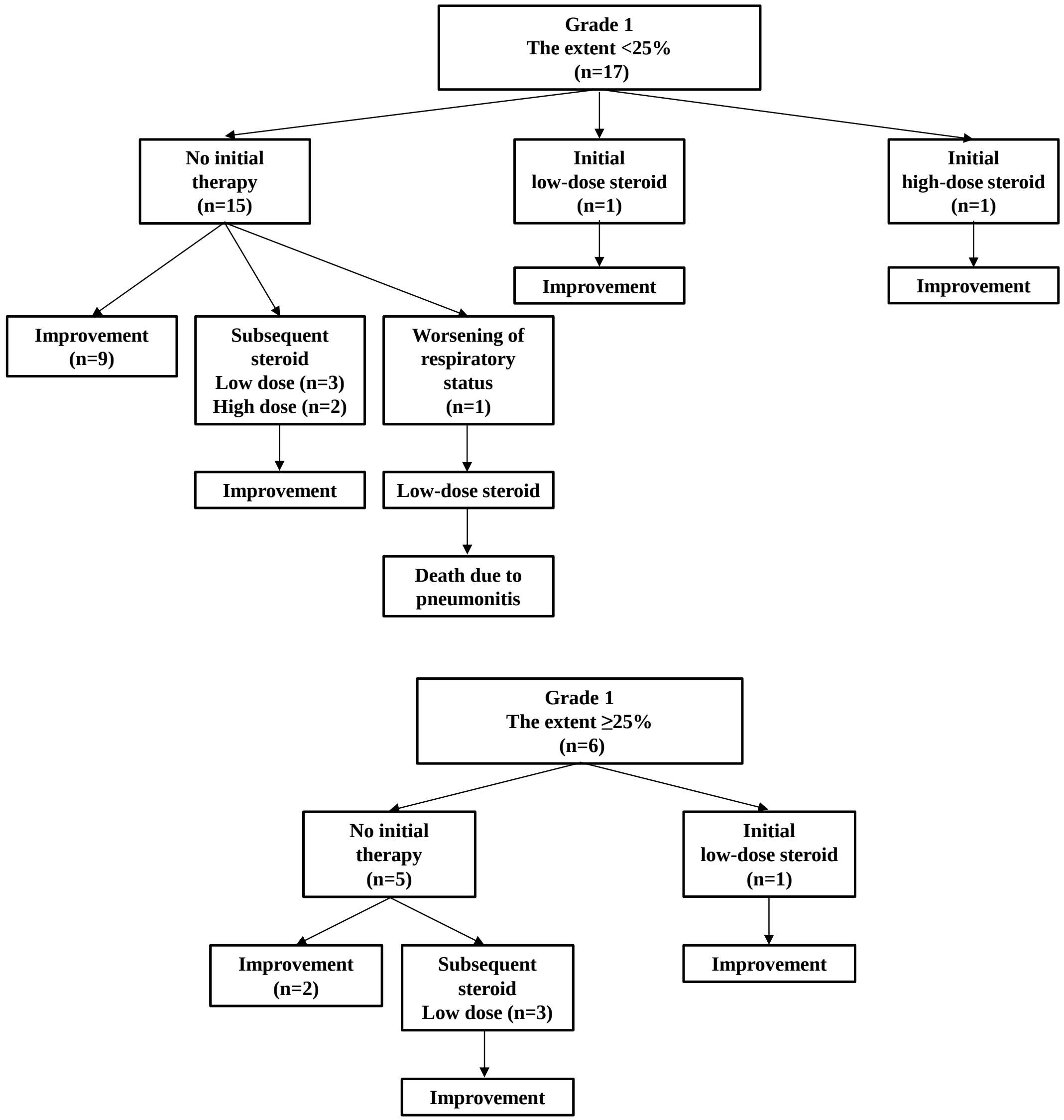

Supplementary Figure S1b

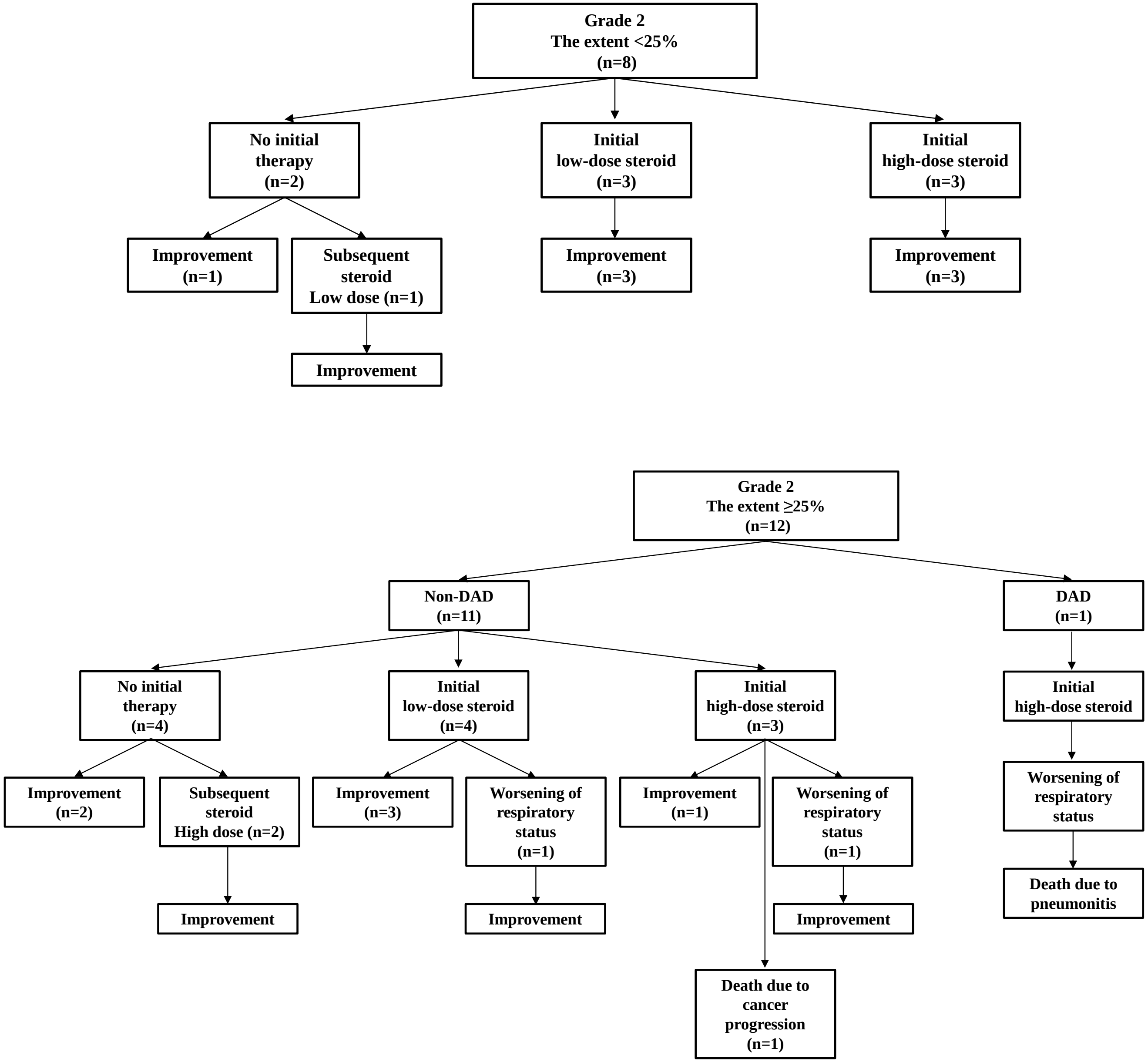

Supplementary Figure S1c

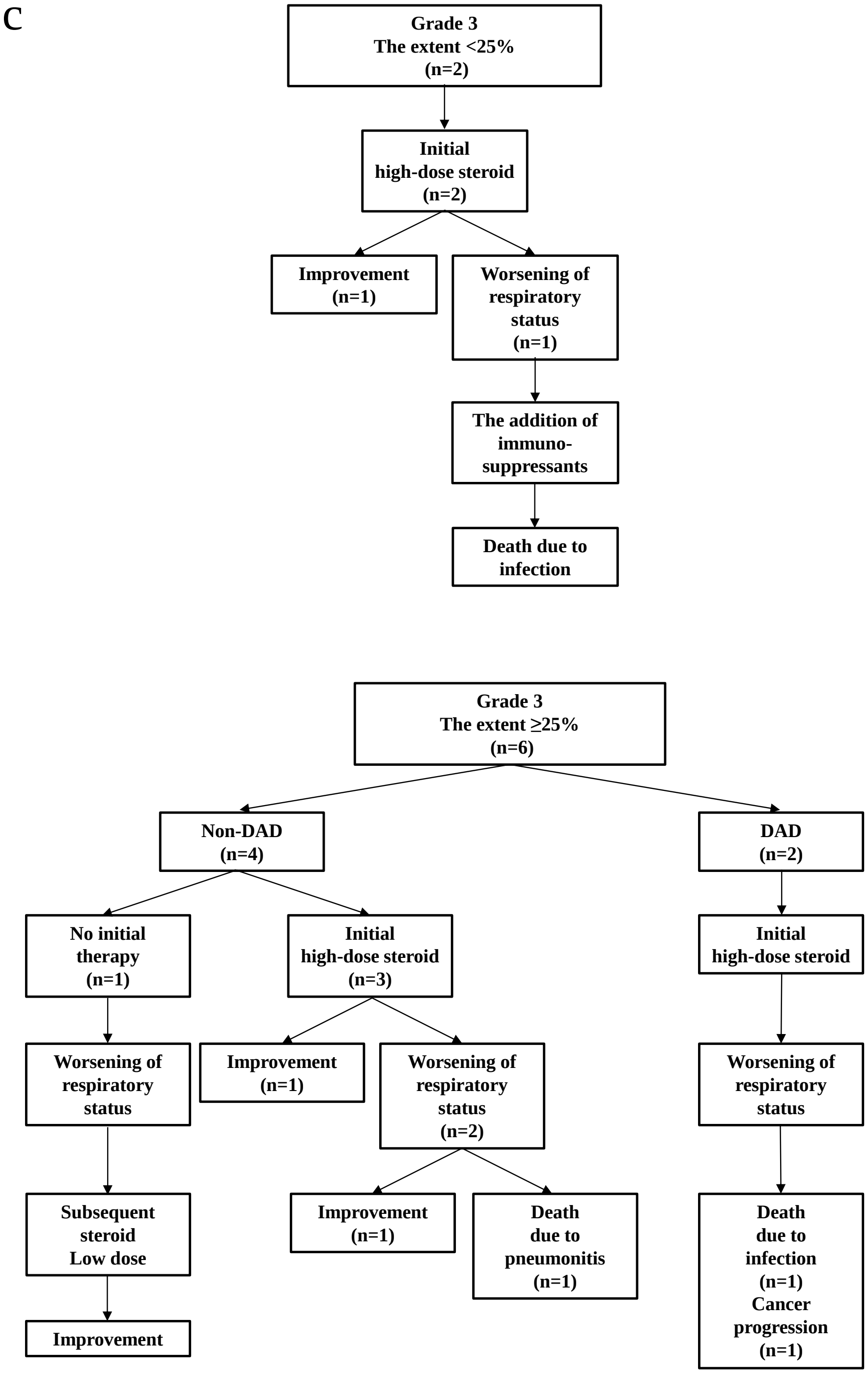

Supplementary Figure S1d

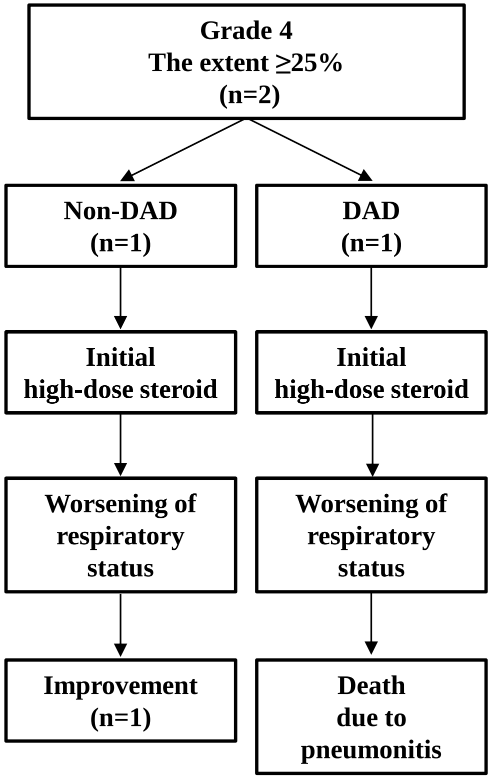

Supplementary Figure S2.
Kaplan–Meier survival curves for survival after the diagnosis of pneumonitis.

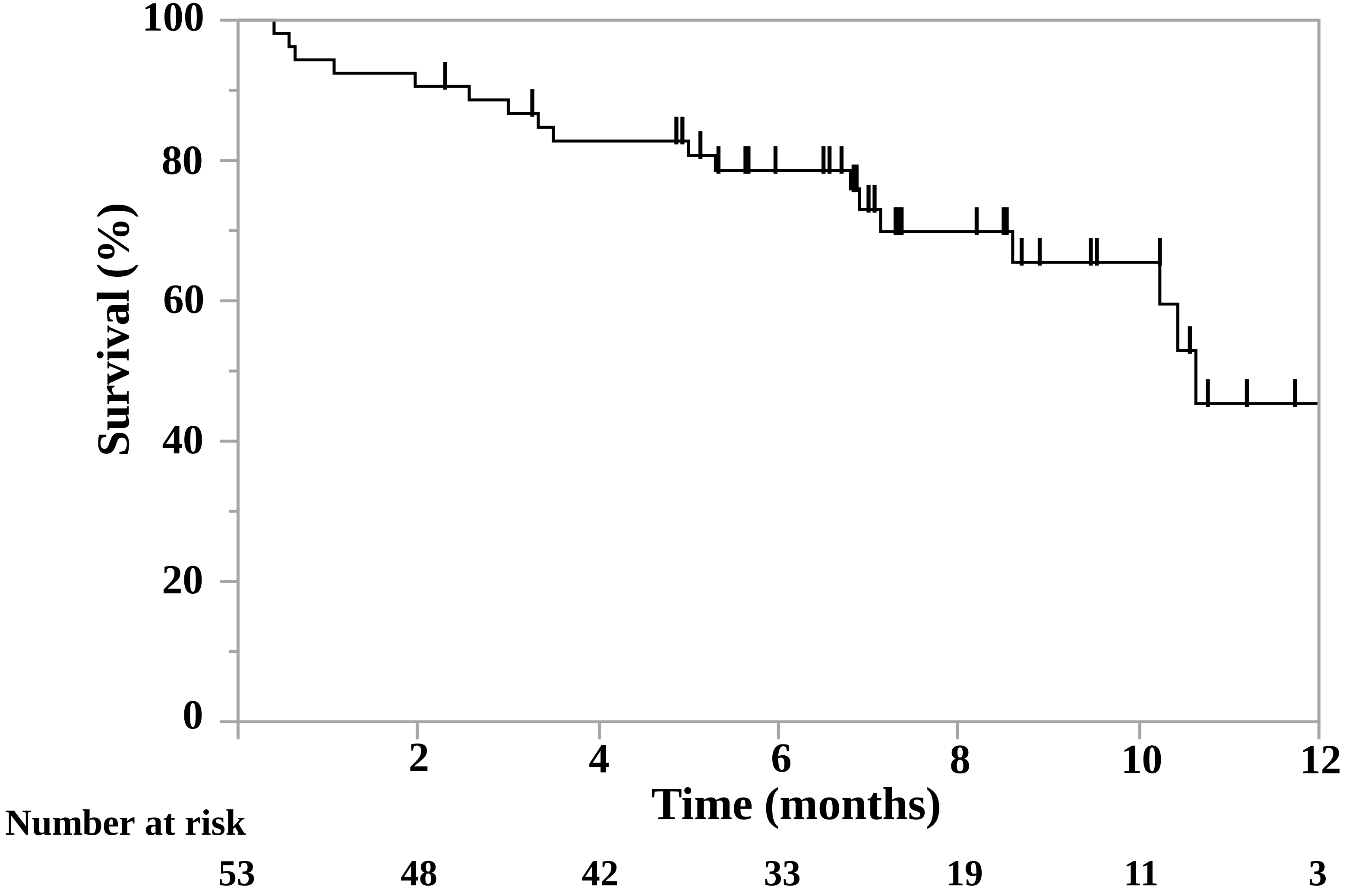

Supplementary Figure S3. Kaplan–Meier survival curves of survival stratified by the Common Terminology Criteria for Adverse Events grade of pneumonitis at diagnosis (a), detailed computed tomography image patterns (b), and categories of the extent of pneumonitis (c).

Supplementary Figure S3a.

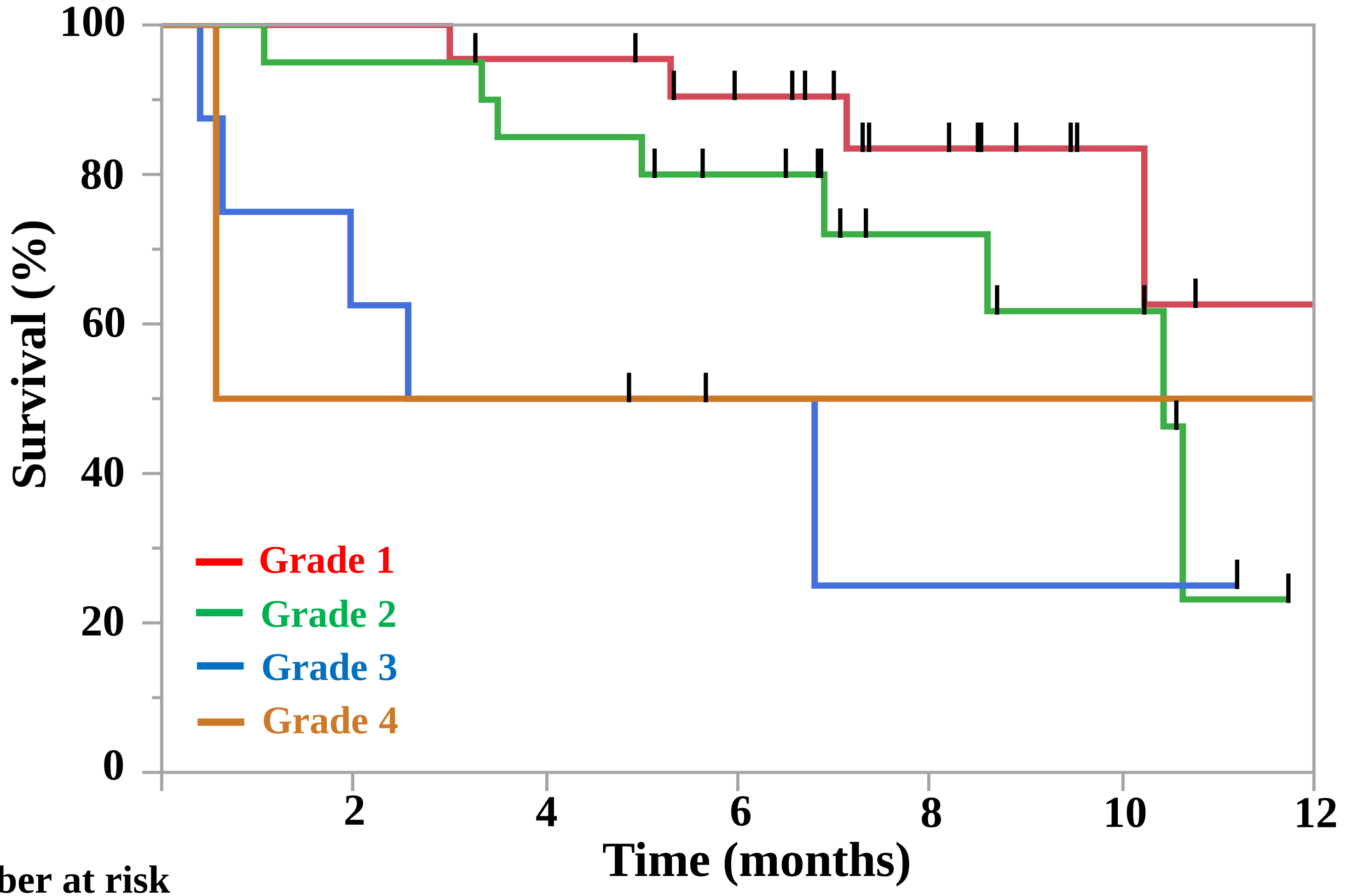

Number at risk

Grade 1	23	23	20	16	10	4	2
Grade 2	20	19	17	14	7	5	0
Grade 3	8	5	4	2	1	1	0
Grade 4	2	1	1	1	1	1	1

Supplementary Figure 3b

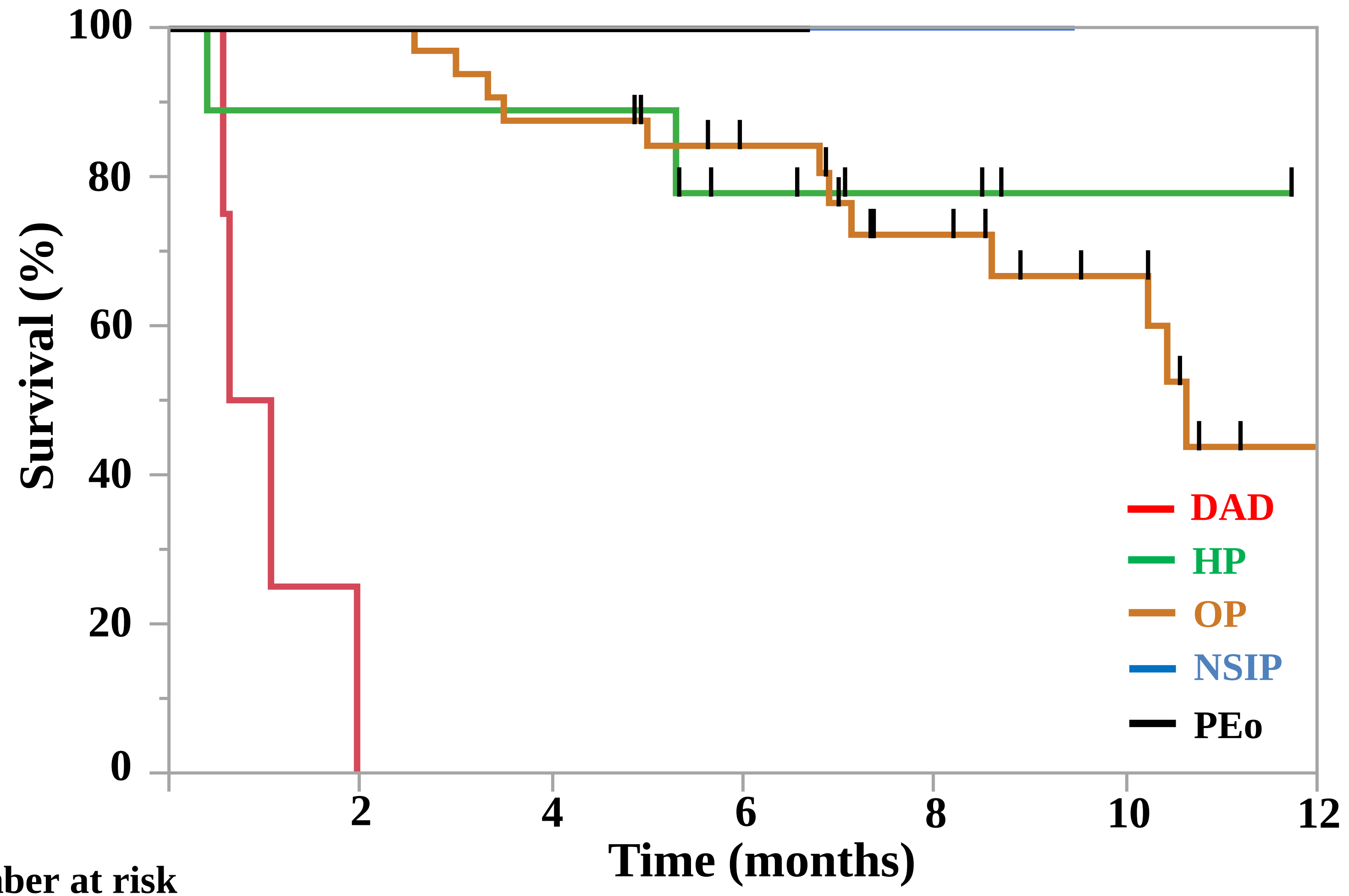

Number at risk

DAD	4	0	0	0	0	0	0
HP	9	8	8	5	3	1	0
OP	33	33	28	23	15	10	3
NSIP	5	5	5	4	1	0	0
PEo	2	2	1	1	0	0	0

Supplementary Figure 3c

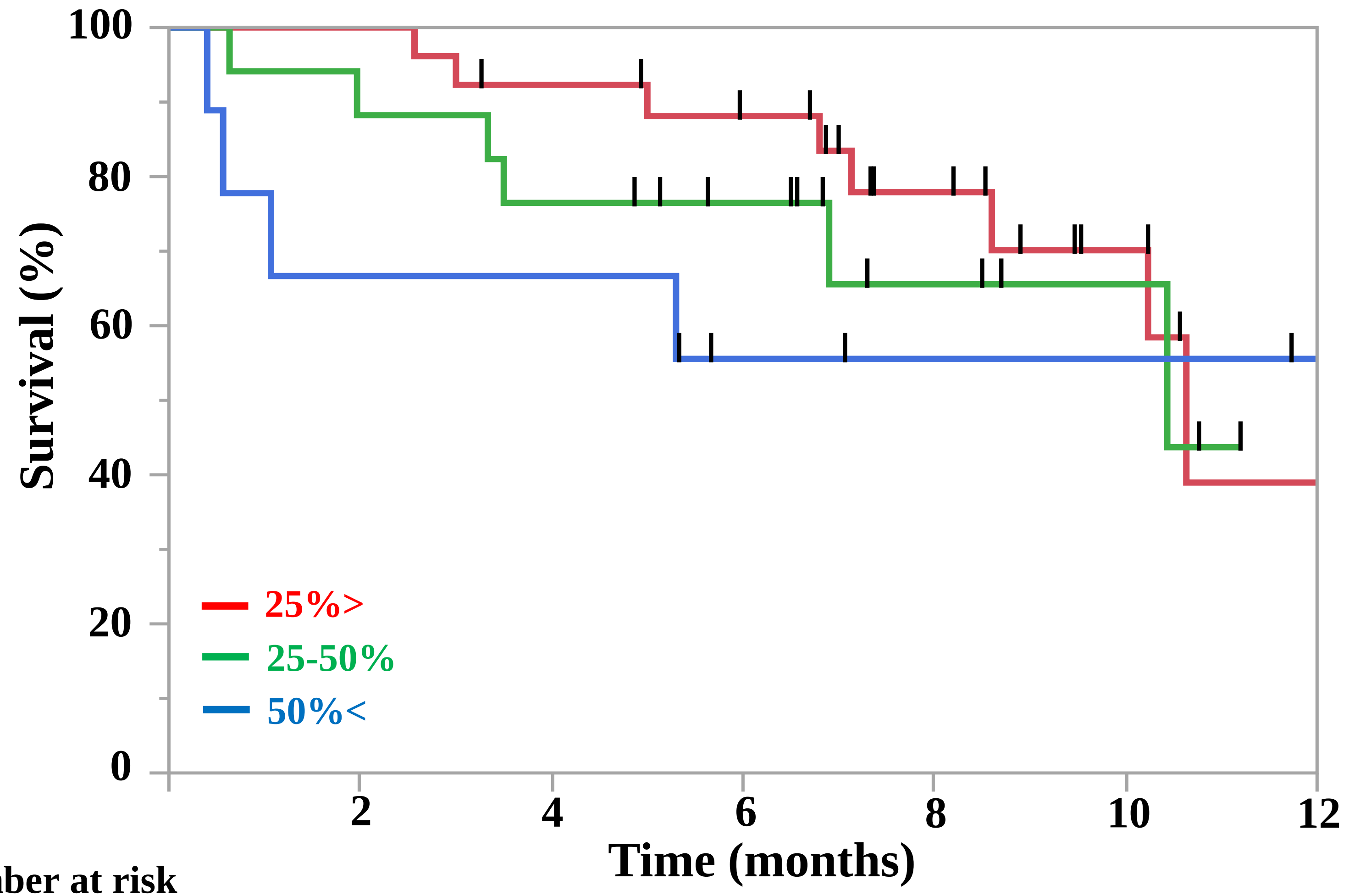

Number at risk

25%>	27	27	23	20	12	6	2
25-50%	17	15	13	10	5	3	0
50%<	9	6	6	3	2	2	1
